# Platelet Induced Functional Alteration of CD4^+^ and CD8^+^ T Cells in HNSCC

**DOI:** 10.3390/ijms21207507

**Published:** 2020-10-12

**Authors:** Christina Polasky, Franziska Wendt, Ralph Pries, Barbara Wollenberg

**Affiliations:** 1 Department of Otorhinolaryngology, University Hospital of Schleswig-Holstein, 23538 Lübeck, Germany; Franziska.Wendt@med.uni-rostock.de (F.W.); ralph.pries@uksh.de (R.P.); 2 Department of Otorhinolaryngology, University Hospital MRI, Technical University, 81675 München, Germany; barbara.wollenberg@tum.de

**Keywords:** platelets, T cells, PD-1/PD-L1, immune regulation, HNSCC

## Abstract

Platelets (PLT) are the second most abundant cell type in human blood and exert various immune-regulatory functions under both physiological and pathological conditions. In fact, immune cell regulation via platelets has been demonstrated in several studies within the past decade. However, the exact mechanisms behind T cell regulation remain poorly understood. We questioned whether the formation of aggregates of platelets and T cells has an impact on T-cell functions. In the present study, we stimulated PBMC cultures with anti-CD3 and anti-CD28 mABs and cultured them at a PLT: PBMC ratio of 1:1 or 100:1. After 24, 48, and 72 h, PD-1, PD-L1 expression, and proliferation were analyzed on T cells using flow cytometry. Cytokine production was measured in PHA stimulated CD4 cells after 6 h. We found a significant platelet-mediated decrease in PD-1 and PD-L1 expression, proliferation, as well as IFN-γ and TNF-α production. Perturbations also at least partially remained after spatial separation of PLTs from PBMCs in Transwell-assays. T cell-platelet aggregates showed similar levels of activation markers, proliferation, and secreted cytokines as their non-complexed counterparts. Results indicate a platelet mediated regulation of T cells via direct and indirect contact, but only mediocre effects of the complex formation itself.

## 1. Introduction

Activation of a T cell is achieved by T cell receptor engagement and a co-stimulatory signal via CD28 [1,2] and characterized by the expression of various surface markers including CD69, CD25 (IL-2Rα chain) [3], as well as the immune checkpoint molecules PD-1 (programmed death-1) and PD-L1 (programmed death ligand-1) [4]. Furthermore, activated T cells start clonal expansion in response to antigen stimulation and produce a vast range of cytokines [5,6,7]. Depending on the co-stimulatory cytokine milieu these include pro-inflammatory IFNs (IFN-α, IFN-γ), TNF-α, and various interleukins. Co-stimulatory molecules are crucial for an adequate T-cell response and include CD27, CD28, GITR, and OX40 [8]. Co-inhibitory signals, such as PD1, PD-L1, and CTLA-4, provide a regulatory mechanism to dampen immune responses [9]. The PD-1/PD-L1 pathway directly contributes to T cell exhaustion and is overly active during chronic infections and in the immunosuppressive tumor microenvironment [10,11]. PD-1 is expressed inducibly on peripheral CD4+ and CD8+ T cells, NK cells, B cells, and monocytes, as well as upon activation on certain dendritic cell (DC) subsets [12]. PD-1 expression on T cells is upregulated by T cell receptor signaling and remains elevated during persistent antigen stimulation [13,14,15]. Expression levels are also found elevated on non-functional, or so-called exhausted T cells in states of chronic viral infections or malignancies [16,17]. PD-L1 is constitutively expressed on endothelial cells, T cells, B cells, DCs, and macrophages and is upregulated even more upon their respective activation [18]. The expression is further regulated by the inflammatory cytokine milieu. IL-2, IL-7, and IL-15 are crucial for T cell survival but also upregulate PD-1 and PD-L1 expression [4]. Type 1 and type 2 interferons and TNF-α also induce PD-L1 expression on T and B cells, endothelial cells, and epithelial cells [12]. A second mechanism regulating T cell response is via regulatory immune cells such as MDSCs, Tregs, or platelets.

Platelets are well-known mediators of thrombosis and hemostasis, but it became increasingly evident, that they actively participate in inflammation and immune responses and therefore play a vital role in the innate immune system [19,20,21]. In humans, platelets account for 150,000–400,000 cells/µl blood and are thus the second most abundant cell type in the blood, hinting their enormous immune-regulatory potential [22,23]. Platelets secrete numerous inflammatory mediators such as adhesion molecules, small molecules, chemokines, and cytokines, that recruit and activate leukocytes at the side of injury [24]. They also exert a regulatory impact by direct contact with immune cells. The binding of activated platelets to leukocytes stimulates cytokine release, oxidative bursts, and the formation of extracellular traps (NETs) [25]. Aggregate formation between platelets and monocytes or neutrophils has been repeatedly described in the context of numerous inflammatory and cardiovascular diseases [26,27,28,29]. There is also evidence for the binding of platelets to lymphocytes [30,31]. Although the functional implication of leukocyte-platelet aggregates (LPA) remains unclear, the aggregate formation with immune cells contributes to the regulation of innate immune responses and is predominantly mediated by CD40 or CD62P (P-Selectin) ligation [32,33]. Platelets binding to T lymphocytes by Gp2b3a, CD154, or CD11b facilitate lymphocyte traveling and adhesion to sites of inflammation [34,35]. Loose co-cultures of platelets induce a decrease in T-cell proliferation, expression of activation markers CD25, PD-L1, and SLAM, as well as the production of IL-17, INF-γ, and TNF-α and an increase in IL-10-expressison [30,36]. It has also been shown that platelets promote a Treg phenotype by selectively enhance the proliferation of FoxP3+ subsets [37].

Platelets are also known to play a crucial role in the development and progression of cancer. High platelet counts are associated with poor prognosis in various cancer types [38,39,40,41,42,43]. Tumor cells interact with platelets via several signaling molecules including ADP and thrombin, which are potent agonists in platelet activation. Upon activation, platelets release many factors, that modulate the tumor microenvironment [44]. Platelets also have been found to enhance epithelial-to-mesenchymal–transition, tumor cell survival in the circulation, extravasation, and colonization at the secondary site. Activated platelets secrete numerous inflammatory molecules from α-granules, which may, depending on the context, promote angiogenesis and immune evasion [45,46].

Here, we try to unravel the impact of platelets on T-cell activation, proliferation, and cytokine production, taking a special interest in platelet-T cell-aggregates. We also addressed the question, whether platelet-mediated T-cell regulation differs in head and neck cancer patients.

## 2. Results

### 2.1. Aggregate Formation Depends on the PLT:PBMC Ratio and Occurs More Frequent in PBMC of Healthy Donors

**Figure 1:**
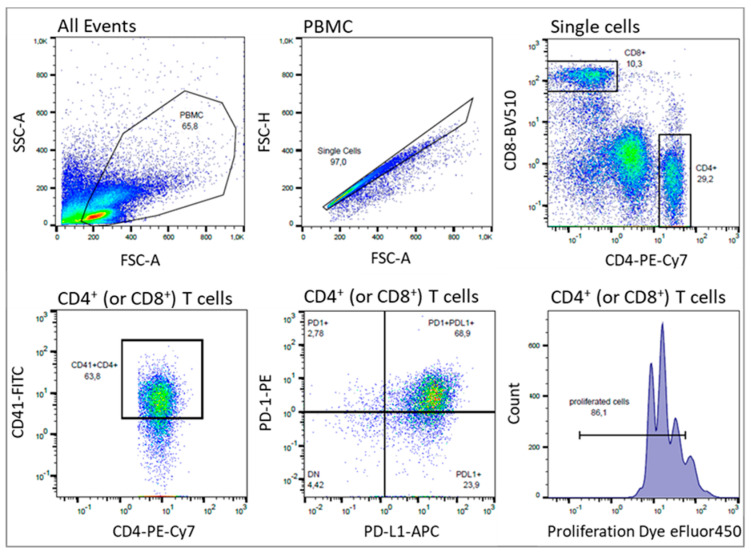
Gating strategy for the flow cytometric analysis of human T cells, platelets and their subpopulations. A single-cell suspension was stained with fluorochrome-conjugated antibodies against CD4-PE-Cy7, CD8-BV-510, PD-1-PE, PD-L1-APC, and CD41-FITC. Furthermore, freshly isolated PBMC were stained with Cell Proliferation Dye eFluor 450 before starting of culture. Data were collected with a MACSQuant flow cytometer and analyzed with FlowJo Software. Lymphocytes were identified by their scatter properties (FSC-A × SSC-A plot) and doublets were excluded by gating on FSC-A × FSC-H. CD4+ or CD8+ cells represent T-cell subsets. Expression of PD-1, PD-L1 and proliferation was investigated on CD4+ as well as CD8+ T cells, but is representatively shown only for CD4. Platelet-T cell aggregate formation was determined by the positivity of the platelet-specific marker CD41 on T cells.

**Figure 2:**
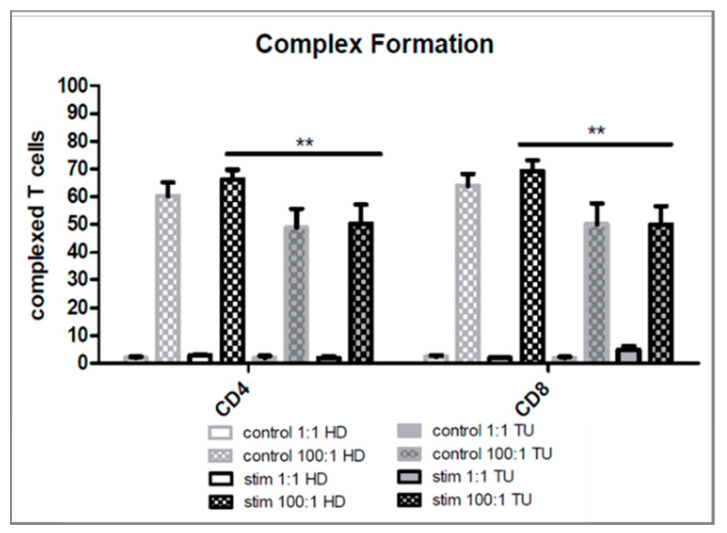
Aggregate Formation of platelets and T cells analyzed by flow cytometry. Percentage of complexed (CD41+) CD4+ or CD8+ T cells in unstimulated or CD3/CD28 stimulated samples from healthy donors (HD) and HNSCC patients (TU) after 24 h. Bars show mean + SEM of results from 20 healthy donors or 7 HNSCC patients. ** *p* ≤ 0.01; two-tailed paired Students’s *t*-test.

We first studied the aggregate formation of platelets with lymphocyte subsets in-vitro (Figure 1). The addition of platelets to PBMC cultures at a physiological blood ratio of 100 platelets per 1 leukocyte (100:1) led to extensive aggregate formation between CD4+ and CD8+ T cells and platelets under unstimulated conditions after 24 h whereas PBMC at a ratio of 1:1 remained non-complexed (Figure 2). Aggregate formation remained at a steady state during the culture period of 72 h. Simultaneous stimulation of PBMC with anti-CD3 and anti-CD28 antibodies led to an additional slight increase of aggregate formation between platelets and T cells from healthy donors. Overall, co-cultures from HNSCC patients showed significantly less complex formation of CD4+ and CD8+ T cells with platelets compared to co-cultures from healthy donors (Figure 2).

### 2.2. Platelets Inhibit TCR-Triggered PD-1/PD-L1 Expression on T Cells in PBMC from Healthy Donors

**Figure 3:**
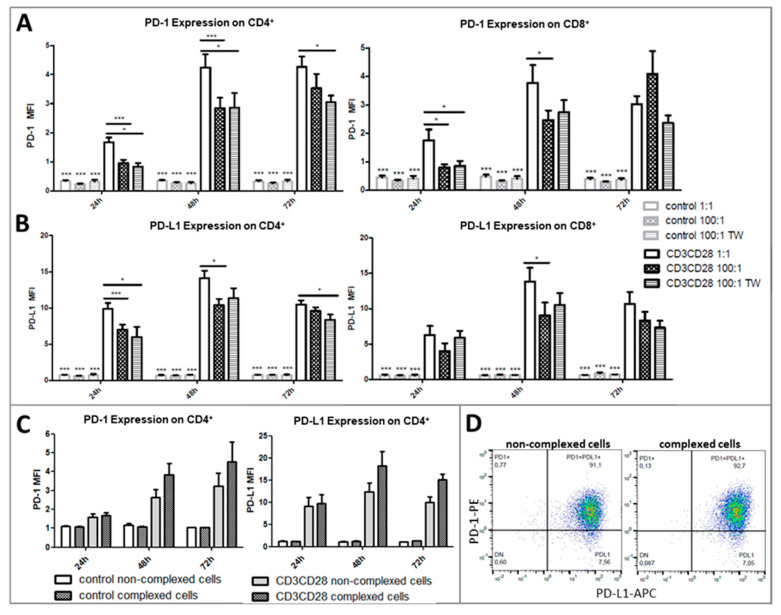
Flow cytometric analysis of the PD-1 and PD-L1 expression levels on CD4+ or CD8+ T cells after 24, 48, and 72 h of culture. Cells were left untreated or stimulated with anti-CD3 and anti-CD28 antibody and cultured in a PLT:PBMC ratio of 1:1, 100:1 (direct contact) or 100:1 in Transwell Assays (TW). (**A**,**B**) PD-1 and PD-L1 expression intensity (MFI) of CD4+ or CD8+ T cells. Bars show mean + SEM of results from 20 healthy donors. (**C**) PD-1 and PD-L1 expression intensity of complexed or non-complexed CD4+ T cells from stimulated or unstimulated cultures. Bars show mean + SEM of results from 6 healthy donors. * *p* ≤ 0.05; *** *p* ≤ 0.001; 1-way Anova with Tukey’s multiple comparisons test. (**D**) Flow cytometric analysis of PD-1 and PD-L1 on complexed and non-complexed CD4+ T cells of one representative sample.

We questioned whether the addition of platelets at a ratio of 100:1 has an impact on the TCR mediated activation of CD4+ and CD8+ T cells. Therefore, we measured the expression level of the T-cell activation markers PD-1 and PD-L1 on CD4+ and CD8+ T cells after 24, 48, and 72 h of culture. In unstimulated control-wells, neither PD-1 or PD-L1 expression was detected on CD4+ and CD8+ T cells, as seen by very low MFI values, that were at similar levels as in unstained control samples. The addition of platelets with direct contact in contrast to Transwell cultures also had no detectable effect on the expression of either marker. A TCR-triggered activation by anti-CD3 and anti-CD28 antibodies induced a strong and significant increase of PD-1 and PD-L1 expression on CD4+ and CD8+ T cells (Figure 3A). Upon addition of platelets to the PBMC cultures, the PD-1 expression on CD4+ and CD8+ T cells decreased significantly after 24 and 48 h (Figure 3A). Remarkably, the spatial separation of PBMCs and platelets using a 0.4 µm pore membrane also led to platelet-driven downregulation of PD-1 expression on T cells.

Similar to PD-1, the PD-L1 expression was also significantly down-regulated by platelets on CD4+ T cells after 24 and 48 h, and on CD8+ T cells after 48 h (Figure 3B). A spatial separation equally reduced the expression of PD-L1. Notably, the platelet covered CD4+ or CD8+ T cells did not show a decrease in activation markers, when compared to uncovered cells (Figure 3C,D), suggesting that aggregate formation itself is not responsible for platelet driven T-cell regulation. In contrast, platelet covered CD4+ T cells always showed a higher PD-1 or PD-L1 expression than non-complexed cells by tendency (Figure 3C).

**Figure 4:**
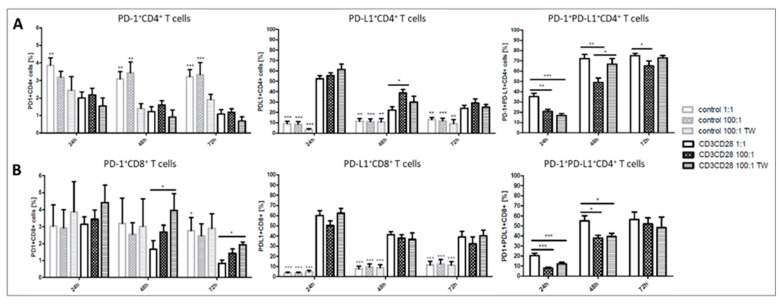
Flow cytometric analysis of the percentage of PD-1 and PD-L1 positive CD4+ (**A**) or CD8+ (**B**) T cells from healthy donors after 24, 48, and 72 h of culture. Cells were left untreated or stimulated with anti-CD3 and anti-CD28 antibody and cultured in a PLT: PBMC ratio of 1:1, 100:1 (direct contact), or 100:1 in Transwell Assays (TW). The number of PD-1+PD-L1+ T cells was not evaluable in unstimulated cultures and are not shown. Bars show mean + SEM of results from 20 healthy donors. * *p* ≤ 0.05; ** *p* ≤ 0.01; *** *p* ≤ 0.001; 1-way Anova with Tukey’s multiple comparisons test.

In line with the effects on the PD-1 and PD-L1 expression levels, the amount of PD-L1+, as well as PD-1+PD-L1+ CD4+ or CD8+ T cells, increased significantly upon TCR specific activation, whereas the percentage of PD-1 single positive cells decreased slightly (Figure 4A,B). Interestingly, most T cells appeared PD-1 and PD-L1 double positive in response to TCR-mediated stimulation. An addition of platelets at a ratio of 100:1 in stimulated cultures led to a significant decrease of PD-1+PD-L1+ CD4+ cells after 24, 48, and 72 h and of CD8+ cells after 24 and 48 h. CD4+ and CD8+ T cells from Transwell inserts showed the same decrease of double-positive cells as unseparated co-cultures after 24 h (Figure 4). The percentages of PD-1 as well as PD-L1 single positive T cells were not significantly altered by platelets.

### 2.3. Platelets Inhibit T-Cell Proliferation in PBMC from Healthy Donors

**Figure 5:**
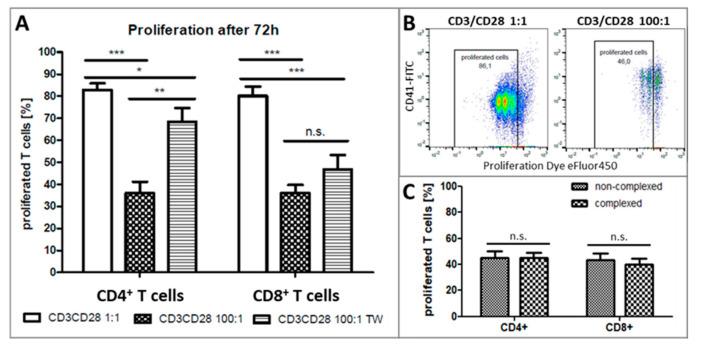
Proliferation of T cells from healthy donors after 72 h anti-CD3 and anti-CD28 stimulated culture. (**A**) Flow cytometric analysis of the percentage of proliferated CD4+ or CD8+ T cells. Cells were cultured in a PLT: PBMC ratio of 1:1, 100:1 (direct contact) or 100:1 in Transwell Assays (TW). Bars depict mean + SEM of results from 20 healthy donors. n.s. *p* ≥ 0.05; * *p* ≤ 0.05; ** *p* ≤ 0.01; *** *p* ≤ 0.001; 1-way Anova with Tukey’s multiple comparisons test. (**B**) One representative example of the proliferative activity of CD4+ T cells from cultures with platelets at a ratio of 1:1 and 100:1. CD41+ complexed CD4+ T cells were also proliferating, although platelets had an inhibitory effect. (**C**) Percentages of proliferated complexed or non-complexed CD4+ or CD8+ T cells after 27 h stimulated culture at a PLT: PBMC ratio of 100:1. Bars show mean + SEM of results from 6 healthy donors. n.s. *p* ≥ 0.05; two-tailed paired Students’s *t*-test.

The TCR-specific stimulation by anti-CD3 and anti-CD28 monoclonal antibodies induced a successive increase of proliferated CD4+ and CD8+ T cells during the culture reaching a maximum of about 90% after 72 h (Figure 5A) while unstimulated cultures remained mostly not proliferative. In contrast, the T cell proliferation of both subsets was significantly decreased in the presence of platelets at a ratio of 100:1 with direct as well as indirect contact after 72h (Figure 5A,B). The spatial separation of PBMC and platelets could restore the proliferative activity of CD4+ T cells but was still significantly reduced when compared to cultures without platelets (Figure 5A). As opposed to that, CD8+ T-cell proliferation showed no significant difference between cultures with direct contact or with separation by a membrane (Figure 5A). Results hint to different mechanisms of regulation of CD4 or CD8 T-cell proliferation, whereby soluble factors and direct contact may play a key role in this context. Interestingly, T-cell proliferation was not influenced by aggregate formation with platelets as seen by equally proliferating CD41+ complexed T cells and non-aggregated ones (Figure 5C).

### 2.4. Impact of Platelets on Cytokine Production of CD4+ T Cells

We next questioned whether the cytokine production of CD4+ T cells was influenced by platelets. PBMC cultures were stimulated with PHA for 6h in the presence of Golgi Stop reagent and intracellular IFN-γ and TNF-α levels were measured afterwards by flow cytometry. In unstimulated controls almost no cytokine-producing cells were detectable. The addition of platelets to unstimulated PBMC led to a measurable increase of IFN-γ+CD4+ cells and significantly increased IFN-γ expression compared to cultures without additional platelets.

**Figure 6:**
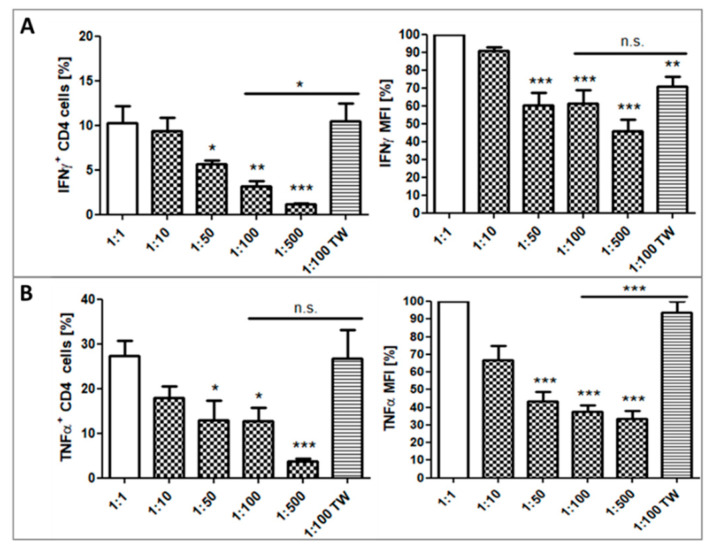
Percentage of IFN-γ (**A**) and TNF-α (**B**) positive CD4+ T cells and Cytokine expression levels (MFI) measured by flow cytometry after PHA stimulated culture for 6 h at a PLT: PBMC ratio of 1:1, 10:1, 50:1, 100:1, 500:1, or 100:1 in Transwell cell culture inserts. Bars show mean + SEM of results from 10 healthy donors. n.s. *p* ≥ 0.05; * *p* ≤ 0.05; ** *p* ≤ 0.01; *** *p* ≤ 0.001; 1-way Anova with Tukey’s multiple comparisons test.

PHA stimulated cells without additional platelets showed a significant increase of IFN-γ+ or TNF-α+ CD4+ cells. The percentage of IFN-γ+CD4+ T cells was significantly reduced proportionally to the increasing ratio of added platelets (50:1, 100:1, and 500:1) in PHA stimulated cultures (Figure 6 left panel). The number of TNF-α+CD4+ T cells was similarly reduced on a significant level at ratios 50:1, 100:1, and 500:1. Furthermore, the expression intensities (MFI) in cytokine positive cells were significantly reduced in the presence of higher numbers of platelets compared to cultures with low platelets (Figure 6 right panel).

To further examine whether the observed inhibitory effect was contact-dependent, platelets, and PBMC at a ratio of 100:1 were also cultured separated by a 0.4 µm pore membrane. CD4+ cells from Transwell inserts showed no decrease of IFN-γ+CD4+ T cells compared to cultures without additional platelets, but the expression intensity of IFN-γ+CD4+ T cells was significantly lower (Figure 6A). In contrast to that, the percentage of TNF-α+CD4+ T cells was not decreased in cultures with Transwell inserts and cells showed also no decrease in the TNF-α expression intensity (Figure 6B). Of note, we observed that CD41+ complexed CD4+ T cells were still IFN-γ+ or TNF-α+ and showed no reduction in cytokine expression intensity compared to non-complexed CD4+ T cells.

### 2.5. Impact of Platelets on PD-1/PD-L1 Expression and Proliferation on T-Cells in PBMC from HNSCC Patients

**Figure 7:**
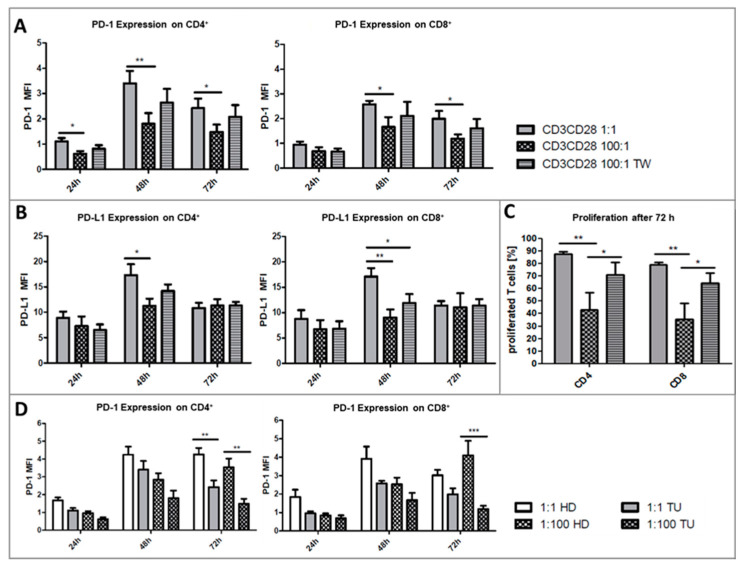
Flow cytometric analysis of the PD-1 (**A**) and PD-L1 (**B**) expression levels on CD4+ or CD8+ T cells from head and neck cancer (HNSCC) patients after 24, 48, and 72 h of culture. Cells were stimulated with anti-CD3 and anti-CD28 antibody and cultured in a PLT: PBMC ratio of 1:1, 100:1 or 100:1 in Transwell Assays. (**C**) Percentage of proliferated CD4+ or CD8+ T cells after 72 h anti-CD3 and anti-CD28 stimulated culture. Bars show always mean + SEM of results from 7 HNSCC patients. * *p* ≤ 0.05; ** *p* ≤ 0.01; 1-way Anova with Tukey’s multiple comparisons test. (**D**) PD-1 expression level on CD4+ or CD8+ T cells from healthy donors vs. HNSCC patients after 24, 48, and 72 h of culture. Cells were stimulated with anti-CD3 and anti-CD28 antibody and cultured in a PLT: PBMC ratio of 1:1 or 100:1. Bars show mean + SEM of results from 20 healthy donors and 7 HNSCC patients. * *p* ≤ 0.05; ** *p* ≤ 0.01; *** *p* ≤ 0.001; 1-way Anova with Tukey’s multiple comparisons test.

The TCR-triggered activation by anti-CD3 and anti-CD28 antibodies induced a strong and significant increase of PD-1 as well as PD-L1 expression on CD4+ and CD8+ T cells from HNSCC patients, which were comparable to healthy donors. PD-1 expression levels were significantly hampered by platelets in co-cultures with direct contact after 24 h (CD4+ T cells), and 48 and 72 h (CD4+ and CD8+ T cells) (Figure 7A). The spatial separation of platelets and PBMCs could partly restore the PD-1 expression on CD4+ and CD8+ T cells but was not significantly changed. PD-L1 expression on T cells was likewise induced after stimulation, but addition of platelets at a ratio of 100:1 hardly altered the intensity of the PD-L1 expression (Figure 7B).

The proliferation of T cells was significantly inhibited by platelets at a ratio of 100:1 after 72 h (Figure 7C). Of note, the spatial separation of PBMC and platelets could restore the proliferative activity of CD4+ and CD8+ T cells, ending up on similar levels as cultures with a ratio of 1:1.

The comparison of healthy donors and HNSCC patients revealed lower PD-1 expression levels on CD4+ and CD8+ T cells from cancer patients compared to healthy donors by tendency that were significantly lower only after 72 h (Figure 7D). Of note, CD4+ T cells from HNSCC patients showed a significantly lower PD-1 expression in cultures with a platelet:PBMC ratio of 1:1 and 100:1 after 72 h compared to healthy donors. PD-L1 expression levels were not significantly altered between both groups.

## 3. Discussion

The present study aimed to investigate the impact of platelet-driven T-cell regulation, especially the effect of complex formation on T-cell activation and functions compared to non-complexed T cells. We thereby focused for the first time on the situation in healthy individuals to unravel the general impact of platelets on T-cell functions and compared findings to HNSCC patients.

For a long time, it is known that platelets can drive a regulatory effect on many cells of the immune system [21,22,47,48]. But the actual impact of platelets on immune cell functions remained very controversial. Aggregation of platelets and lymphocytes has been found in chronic infections like HIV [49,50], autoimmune diseases [51], and lung cancer [52]. Binding is mediated by GP2b/3a, CD154, and lymphocyte CD11b and is assumed to be crucial for the recruitment of T cells to sides of inflammation and fine-tuning of their functions [24,53]. However, current literature reveals heterogeneous data reporting either an inhibitory effect of platelets on effector T cells [49], the promotion of regulatory T cell differentiation [30], or even induction of Th1/Th17 cell differentiation [51]. Contradictory results originate mostly from very different experimental setups, the disease, and the T cell subset of interest.

We first focused on the occurrence of complex formation itself. Our study proofs for the first time that complex formation depends on the PLT: PBMC ratio occurring more frequently in PBMC of healthy donors. An increasing ratio of added platelets to isolated PBMCs of the same donor increases the effects induced by platelets. A surprising difference was the lower level of complex formation of platelets from HNSCC patients compared to healthy donors. We expected a higher aggregate formation since platelets of tumor patients were often reported to be more activated and have thereby a higher tendency for complex formation with leukocytes [54,55,56]. A possible explanation could be the already prevailing exhausted phenotype of the T cells characterized by lower responsiveness and expression of activation molecules. More important, in our study complex formation seems to be correlated with the level of T-cell activation and a preferentially higher complex formation with T cells expressing higher levels of PD-1, PD-L1, and CD25 by tendency. We therefore assume that platelets predominantly attach to activated T cells which were likewise assumed in another study [53]. Since T cells of HNSCC patients showed overall lower activation after stimulation, platelet mediated T-cell regulation may be of less importance.

We next examined the influence of platelets by measuring T-cell activation by the help of PD-1/PD-L1 expression, proliferation, and cytokine secretion in T cells in PBMC from healthy donors after TCR triggered activation via CD3/CD28 or PHA, respectively. Unstimulated T cells from healthy donors showed no PD-1/PD-L1 expression which was also not altered by platelets. Results indicate that platelet-driven T-cell regulation is mostly an issue during infections or inflammatory conditions. If T cells or platelets are not activated by any kind of inflammatory stimulus, platelets do neither exert an activating nor inhibitory impact on the herein analyzed parameters, at least under cell culture experimental conditions. The addition of platelets to TCR stimulated T cells in the PBMC bulk clearly revealed inhibitory effects on the PD-1 as well as PD-L1 expression of CD4+ and CD8+ T cells. Transwell assays confirmed that this regulation is mediated mostly by soluble factors as the spatial separation of PBMC and platelets by 0.4 µm cell culture inserts did not show significant differences regarding the PD-1 and PD-L1 expression on CD4+ as well as CD8+ T cells compared to cultures with direct platelet to PBMC contact. The number of PD-1+PD-L1+ T cells on the other hand was differently affected by platelets. For CD4+ T cells, the addition of platelets at a ratio of 100:1 led to a decrease of double-positive cells, whereas percentages of PD-1+PD-L1+ T cells could be restored in separated cultures. In CD8+ T cells, there was also a reduction in platelet-added cultures, but no difference between direct contact or separated co-cultures. For the CD8 subset, we found instead an increase of PD-1 single positive cells in cultures with added platelets (Figure 4B). Our findings reveal new insights into the T-cell activation dynamics and expression patterns of these two checkpoint molecules, which are differentially expressed on CD4+ and CD8+ T cells. The question remains what functional unique features a PD-1 and PD-L1 double-positive T cell possess. PD-L1 is typically expressed on monocytes and other antigen-presenting cells and serves as a negative regulator for activated PD-1+ T cells to control immune responses. PD-L1 on T cells exerts critical functions in controlling self-reactive T cells and limiting pathogenic effector T cell responses [57]. It may be possible that CD4+ T cells acquire these regulatory functions and inhibit their PD-1+ counterparts by themselves. Recent studies concentrate on the existence and increase of PD-L1+ T cells in peripheral blood from patients with different tumor entities [58,59,60,61]. Lately, it has been proposed that PD-L1hi+CD25+CD4+ cells represent regulatory T cells that regulate activated PD-1+CD8+ T cells [62,63].

T cells from HNSCC patients generally showed lower activation levels seen by PD-1 and PD-L1 expression. Nevertheless, cells were stimulable and reacted in similar patterns as T cells from healthy donors. An overall higher PD-L1 expression on CD8+ T cells from tumor patients as reported by other studies could not be validated in our in-vitro settings [64,65]. Platelets from HNSCC patients showed also the same inhibitory effects on T cells compared to those of healthy donors.

In addition to the expression of PD-1/PD-L1, we examined the proliferation and clonal expansion of T cells under the influence of platelets. T cells within the PBMC fraction of healthy donors proliferated significantly after stimulation via CD3/CD28 (Figure 5A). The addition of platelets inhibited T-cell proliferation in PBMC from healthy donors significantly in a cell ratio-dependent manner. Interestingly, CD4+ T cell proliferation was strongly reduced by direct platelet-T cell contact but was mainly restored in separated co-cultures (Figure 5A). As opposed to this, the proliferation of CD8+ T cells was equally reduced in cultures with or without direct platelet contact which indicates a more indirect mechanism of regulation. A possible explanation could also be the lack of pro-inflammatory cytokines like IL-2, which is normally secreted by activated CD4+ T cells. We assume that the cytokine driven proliferation of CD8+ T cells was reduced due to platelet-caused CD4 T-cell inhibition. Carter and colleagues already reported on a higher sensitivity to modulation of CD8+ T cells due to their inability to produce significant levels of IL-2 by themselves [66]. Furthermore, although CD4 T-cell proliferation was mainly regulated by direct platelet-T cell contact, complexed CD4+ T cells showed the same percentage of proliferated cells as their non-complexed counterparts. The same applies for CD8+ T cells. The regulation of T-cell proliferation by platelets seems to happen via different mechanisms, albeit underlying molecular mechanisms remain unclear.

Observation of resting T cells showed a marginal cytokine level. Addition of platelets to those resting cultures induced a significant increase of IFN-γ+CD4+ cells. On the other hand, TNF-α production was not affected by platelets. This observation might be due to the release of serotonin from dense granules of activated platelets, which is an extremely potent inhibitor of TNF-α secretion and simultaneously activator of IL-2, IL-16, and IFN-γ secretion from T cells [62,67]. Stimulation by PHA massively induced IFN-γ and TNF-α expression in CD4+ T cells. Again, exposure of those activated cells to platelets led to a significant, ratio-dependent down-regulation of IFN-γ+CD4+ cells and TNF-α+CD4+ cells as well as their cytokine expression levels (Figure 6A,B). Complex formation of T cells had again no effect on the quantitative cytokine secretion of the T cells.

An inhibitory impact of platelets on T cell functions has already been described by Zamora and colleagues in in-vitro studies [30,36]. Similar to our results, they showed a decrease of CD25, PD-L1 and SLAM4 expression, IL-17, IFN-γ, and TNF-α secretion and proliferation of T cells from PBMC cultures with added platelets. In contrast to our data, they reported a reversion of the effects in membrane-separated cultures or upon anti-P-Selectin treatment. On the other hand, they also found a higher expression of activation markers on platelet-complexed cells as opposed to non-complexed cells [36]. We found a decrease of activation markers on CD4+ and CD8+ T cells in the presence of platelets independent of the culture system. Activated platelets secrete plenty of soluble factors that regulate immune cell activation and differentiation. Factors of activated platelets that directly affect CD4 T cells are cytokines, chemokines, platelet-activating factor 4 (PF4), and thromboxane A2 [24,34,68,69]. They can also release IgGs from their α-granules and are a predominant source of the anti-inflammatory cytokine TGF-β [70]. In contrast, T cell functions seem to be regulated predominantly via direct interactions. The expression of pro-inflammatory cytokines such as IFN-γ and TNF-α by CD4 cells was significantly decreased by platelets in a dose-dependent manner, but effects were abolished when cell-cell contact was prevented in Transwell cultures. T-cell proliferation was likewise decreased in the presence of platelets, but could be at least partly restored by inhibition of cell interactions. We assume that the regulatory impact of platelets on T cells is exerted via direct contact and soluble factors or microvesicles. Current data is very heterogeneous and most authors don’t focus on the characterization of aggregated T cells in comparison to non-complexed T cells.

Results from our study clearly revealed a platelet provoked inhibition of T cell activation, proliferation and secretion of certain cytokines in response to in-vitro stimulations, which is exerted by direct contact and soluble factors. However, we found almost no differences in platelet-complexed and non-complexed CD4+ or CD8+ T cells. Results indicate that aggregate formation per se is not responsible for T-cell inhibition or failure of the adaptive immune system but acts more like a fine-tuned regulation system of immune responses. Non-aggregated platelets also drive a major influence on T cells. A more detailed analysis of functional T cell markers, study of the decisive factors on how platelets regulate T cells, as well as the measurement of aggregate formation in-vivo could be future work based on this article.

## 4. Materials and Methods

### 4.1. Blood Collection

All blood donors have signed informed written consent and were informed about the aims of the study and the use of their samples. Blood samples were collected from healthy donors (n = 20) or head and neck cancer (HNSCC) patients (n = 7). 40 mL of blood was drawn by venipuncture into a sodium citrate containing S-Monovette (Sarstedt AG & Co KG; Nümbrecht, Germany).

### 4.2. Platelet and PBMC Isolation from Whole Blood

Human blood platelets were isolated as described previously [71] with slight modifications. In brief, blood was centrifuged for 5 min at 330× *g* without brake to obtain platelet-rich plasma (PRP). The PRP was transferred to a new 50 mL tube and filled up with phosphate-buffered saline (PBS) (PAN Biotech GmbH, Aidenbach, Germany) supplemented with 1mM EDTA (Merck KGaA, Darmstadt, Germany) to reduce leukocyte contamination and centrifuged for 10 min at 250× *g* without brake. The supernatant was completely transferred to a new 50 mL tube without touching the leukocyte pellet and centrifuged at 430× *g* for 15 min to sediment platelets. The platelet pellet was re-suspended in RPMI 1640 Medium (Gibco^®^ Thermo Fisher Scientific Inc., Rockford, IL, USA) containing 10% FCS (Gibco), 1% Pen/Strep (Merck KGaA), and 1% Pyruvate.

PBMC were isolated from the remaining blood fraction after taking off the PRP by density gradient centrifugation in Biocoll (Biochrom GmbH, Berlin, Germany) at 400× *g* for 20 min. The remaining PBS/plasma layer was carefully removed and discarded. The PBMC layer was carefully harvested and transferred to a new 50 mL tube and washed 4 times with PBS followed by slow spin centrifugation at 120× *g* for 10 min to get rid of remaining platelets. The PBMC pellet was finally also re-suspended in RPMI 1640 Medium containing 10% FCS, 1% Pen/Strep, and 1% Pyruvate. For Proliferation analysis, freshly isolated PBMC were re-suspended in PBS and labeled with Cell Proliferation Dye eFluor 450 (Thermo Fisher Scientific Inc., Rockford, IL, USA) according to the manufacturer’s protocol.

### 4.3. Cell Count and Purity Assessment of Isolated Cells by Flow Cytometry

The purity of isolated platelets and PBMCs was assessed by flow cytometry using FSC/SSC characteristics and staining for CD45 and CD41, respectively. Platelet purity was always ≥99%. Platelet contamination of PBMCs was despite washing between 20% and 40%. Cell numbers were determined in the same way by a MACSQuant 10 Flow Cytometry system (Miltenyi Biotec, Bergisch Gladbach, Germany) by measuring 10 µL of the freshly isolated cell suspensions diluted with 90 µL PBS.

### 4.4. Platelet and PBMC Activation and Co-Culture Experiments

PBMCs were co-cultured with Platelets at a physiological blood ratio of 1:100. In some experiments, 1:10, 1:50, and 1:500 ratios were additionally used. Due to platelet contamination of PBMC fractions, a ratio of 1:1 served as control. Cell-culture experiments were performed in 12-well flat-bottom cell culture plates (Sarstedt AG & Co KG, Nürmbrecht, Germany) at 37 °C and 5% CO_2_. In some experiments, a cell culture insert with 0.4 µm pore membrane (Becton Dickinson GmbH, Heidelberg, Germany) was used to study whether platelet mediated effects on T cells were contacted dependent or independent (Transwell insert).

To stimulate PBMC, 12-well plates were coated with anti-CD3 by pre-incubation of the plates with 5 µg/mL antibody in PBS for 2 h. Furthermore, cell cultures were supplemented with soluble anti-CD28 antibody (2 µg/mL) at the start of the culture experiment. Untreated cultures served as a biological control for successful T-cell activation and proliferation.

### 4.5. Investigation of Cytokine Production of CD4+ T Cells

To evaluate cytokine production of CD4+ T cells, PBMCs and platelets were co-cultured in a ratio of 1:1, 1:10, 1:50, 1:100, and 1:500. PBMC were stimulated with 0.5 µg/mL Phytohaemagglutinin (PHA; Merck KGaA, Darmstadt, Germany) for 6 h in the presence of Monensin Solution (Thermo Fisher Scientific Inc., Rockford, IL, USA) to inhibit protein transport and improve cytokine staining. Cells were harvested and stained for CD4, CD41, PD-1, and PD-L1 as described below. Intracellular staining with INF-γ-APC or TNF-α-APC was performed after using the Fixation/Permeabilization Solution Kit (Becton Dickinson, Heidelberg, Germany) according to the manufacturer’s protocol.

Cytokine production of CD4+ T cells from HNSCC patients could not be investigated due to non-sufficient isolated cell numbers of PBMC and platelets from those patients.

### 4.6. Flow Cytometry

Flow cytometry was performed with a MACSQuant 10 flow cytometer (Miltenyi Biotec, Bergisch Gladbach, Germany) and data was analyzed using the FlowJo software version 10.0 (FlowJo, LLC, Ashland, OR, USA). Antibody titrations and compensation was performed in beforehand. To measure T-cell activation, PD1/PD-L1 expression, and T-cell platelet aggregate formation, the following antibodies were used: CD4-PE-Cy7, CD8-BV-510, PD1-PE, PD-L1-APC, CD41-FITC (all from Biolegend, San Diego, CA, USA). T-cell Proliferation was assessed by the decreasing fluorescence intensity of the proliferation-dye. Human TruStrain FcX (BioLegend) was added before staining to avoid unspecific binding of the antibodies. Gating strategy of CD4 and CD8 positive T lymphocytes, as well as PD-1 and PD-L1 positives and proliferated cells, are shown in Figure 1.

### 4.7. Statistical Analysis

Statistical analyses were performed with GraphPad Prism Version 7.0f. The mean and standard error (SEM) are presented. The differences between groups were determined after testing for Gaussian distribution (normality tests), and applying parametric (student’s *t*-test), or non−parametric 1-way ANOVA, Tukey’s multiple comparisons test: *p* < 0.05 (*), *p* < 0.01 (**), and *p* < 0.001 (***). Additional statistical details are given in the respective figure legends, when appropriate.
